# Supplementary material for: Hyaluronan-Induced CD44-iASPP Interaction Affects Fibroblast Migration and Survival
Source: Cancers (Basel). 2023 Feb 8;15(4):1082. doi: 10.3390/cancers15041082 (PMC9954134; doi:10.3390/cancers15041082)
Supplement: Supplementary file 1 [file cancers-15-01082-s001.zip › File S1.pdf]

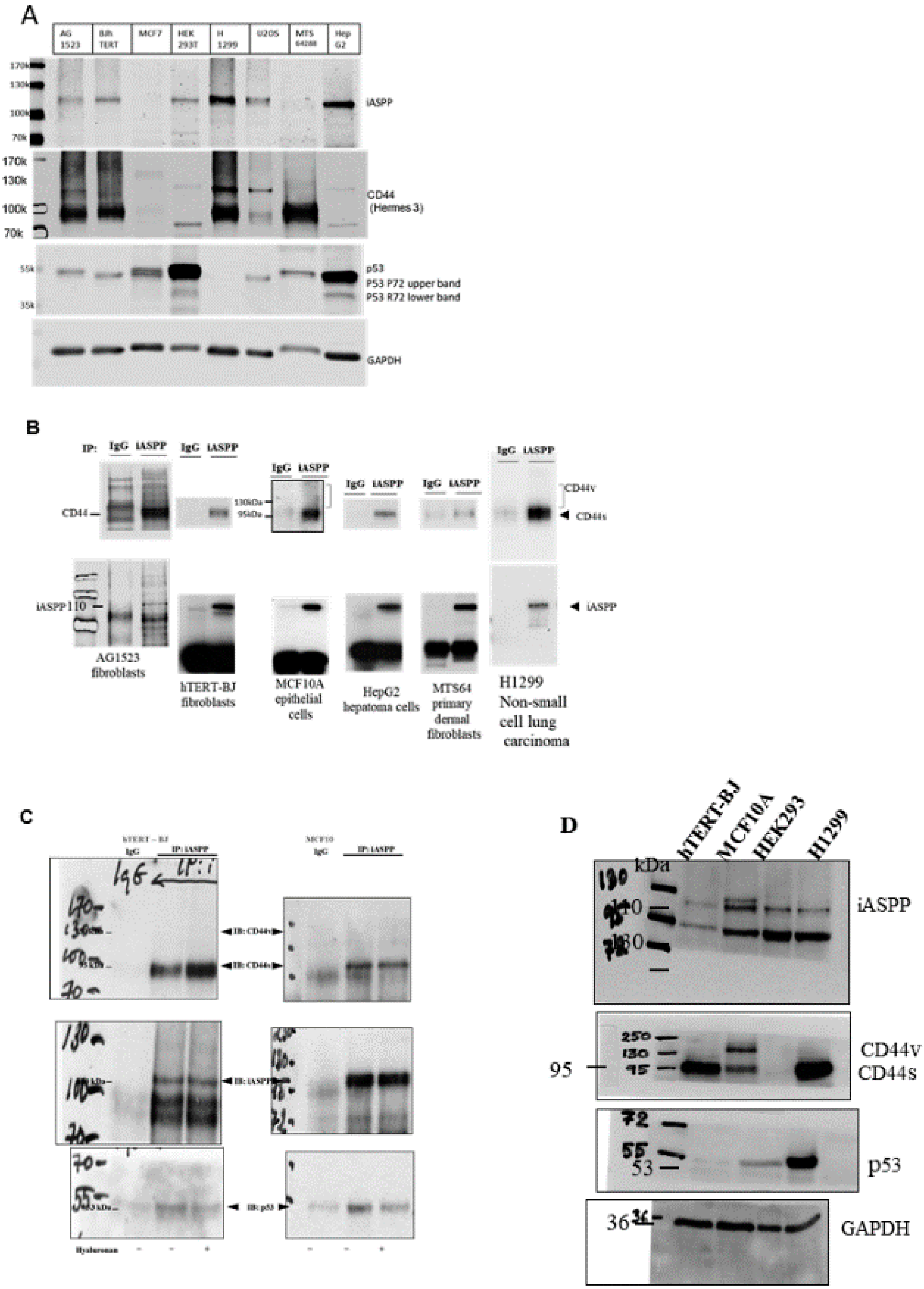

Uncropped blots corresponding to Figure 1

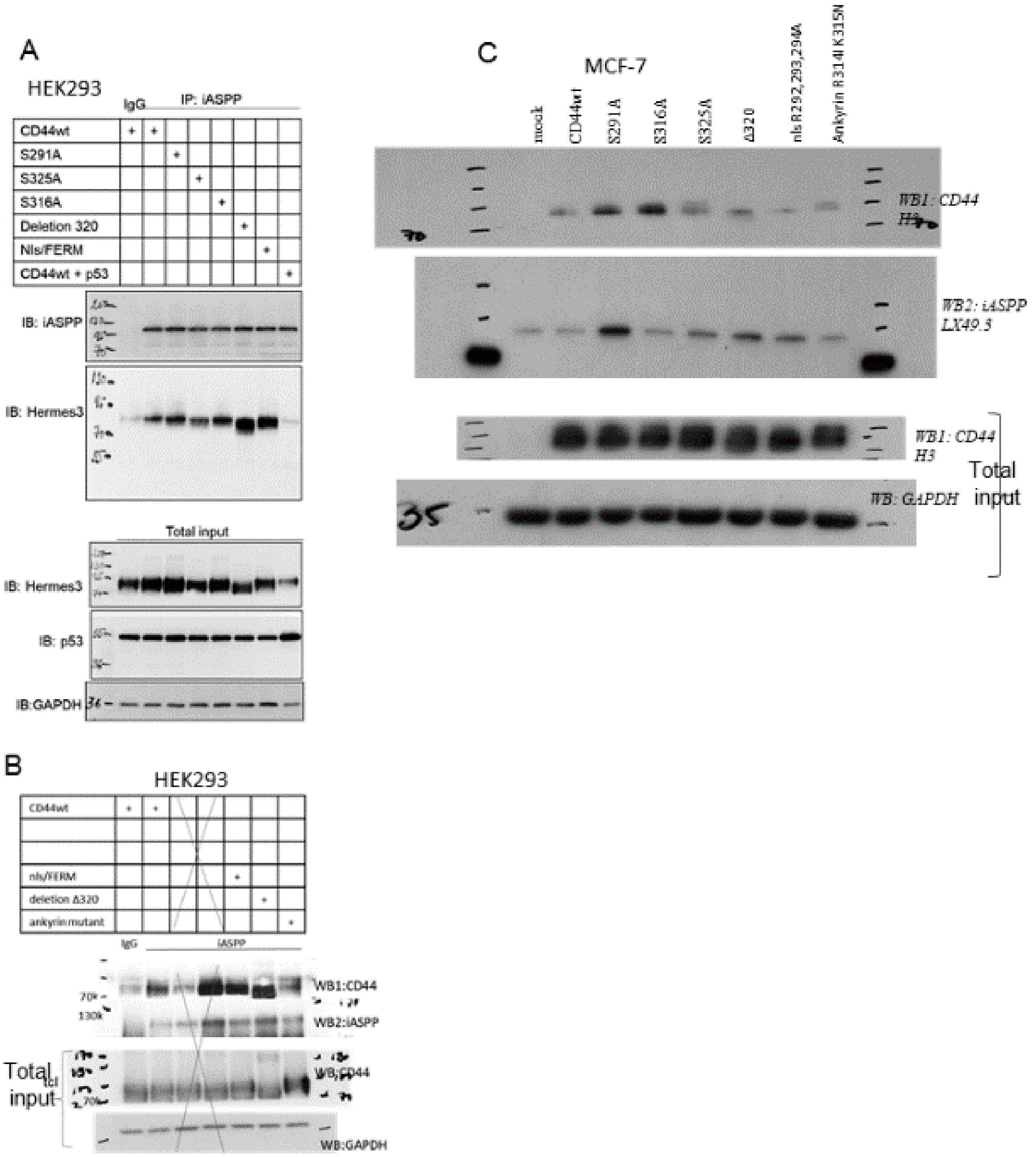

Uncropped blots corresponding to Figure 3

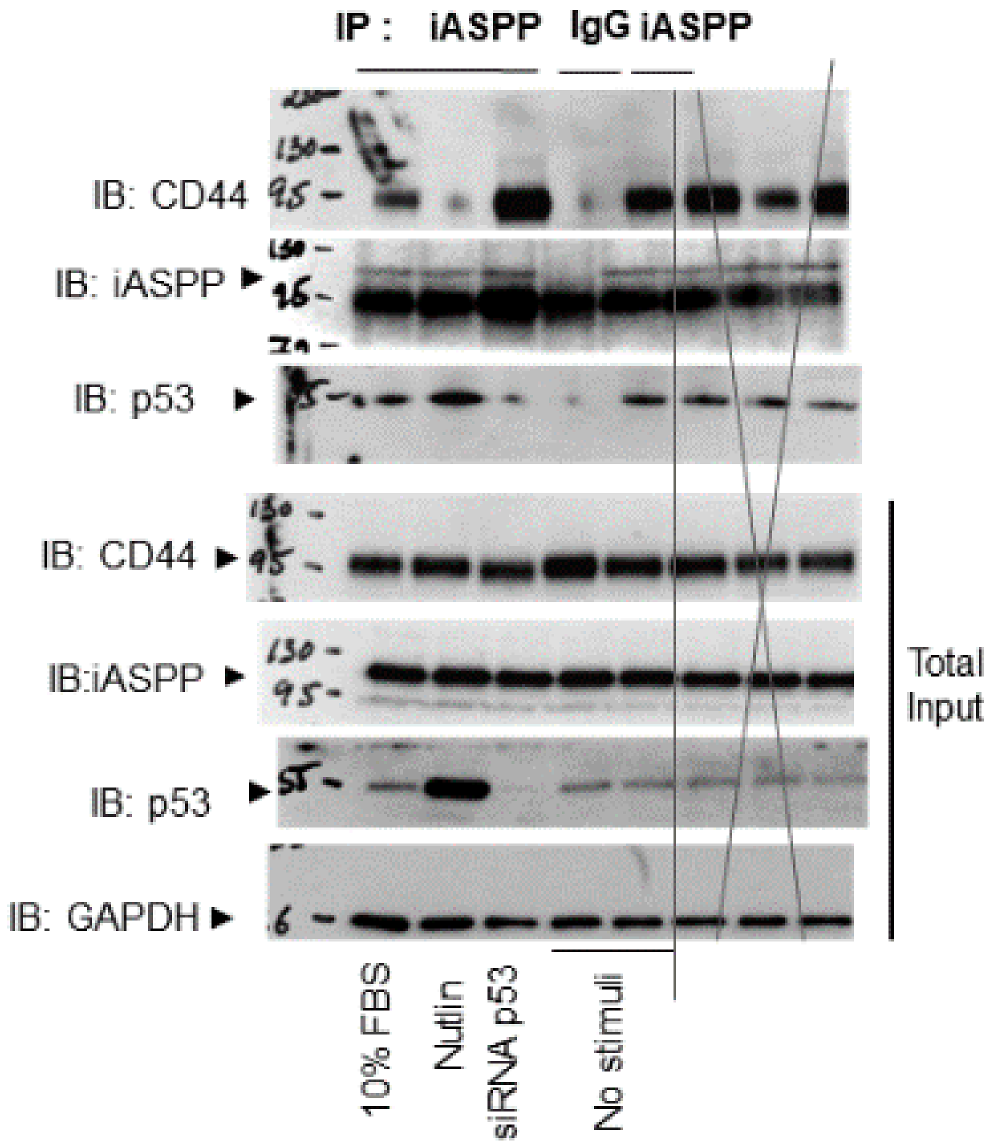

Uncropped blots corresponding to Figure 4

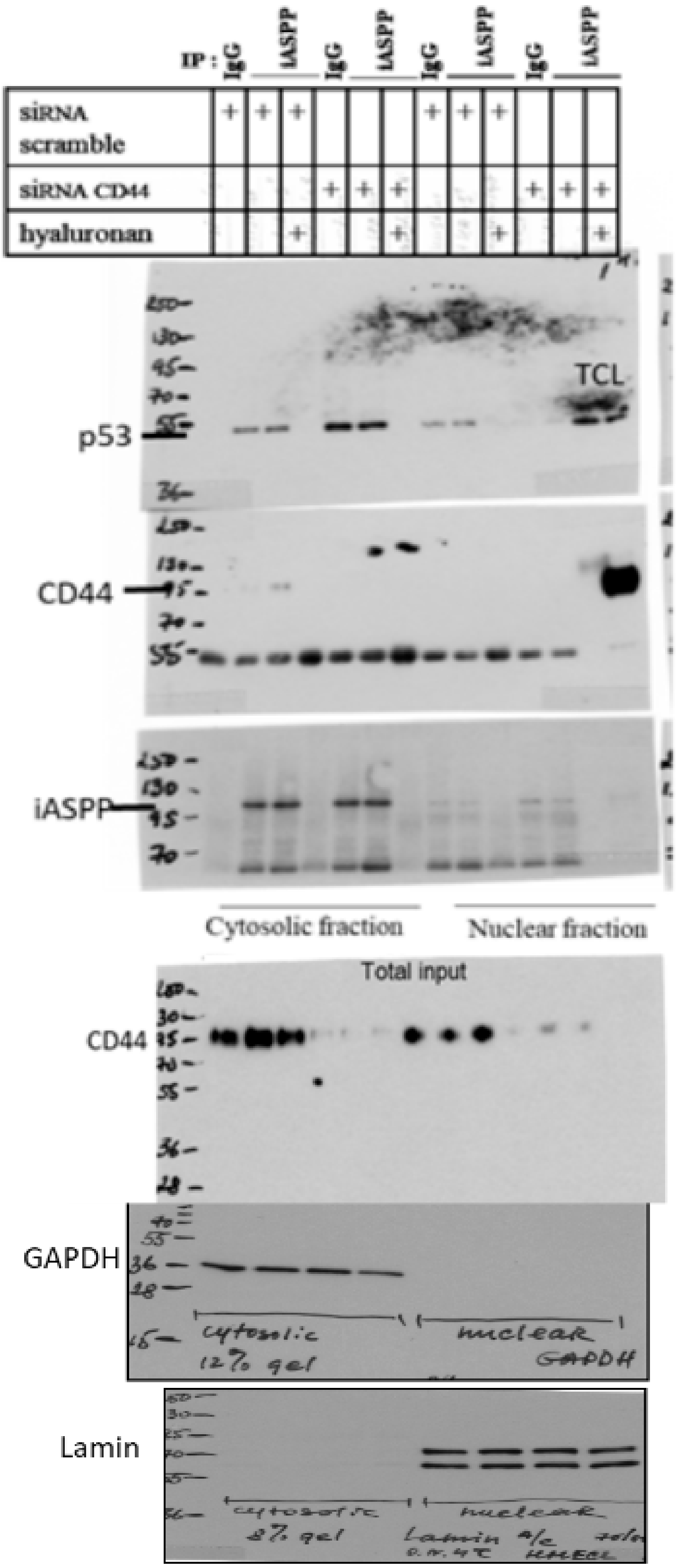

Uncropped blots corresponding to Figure 5B

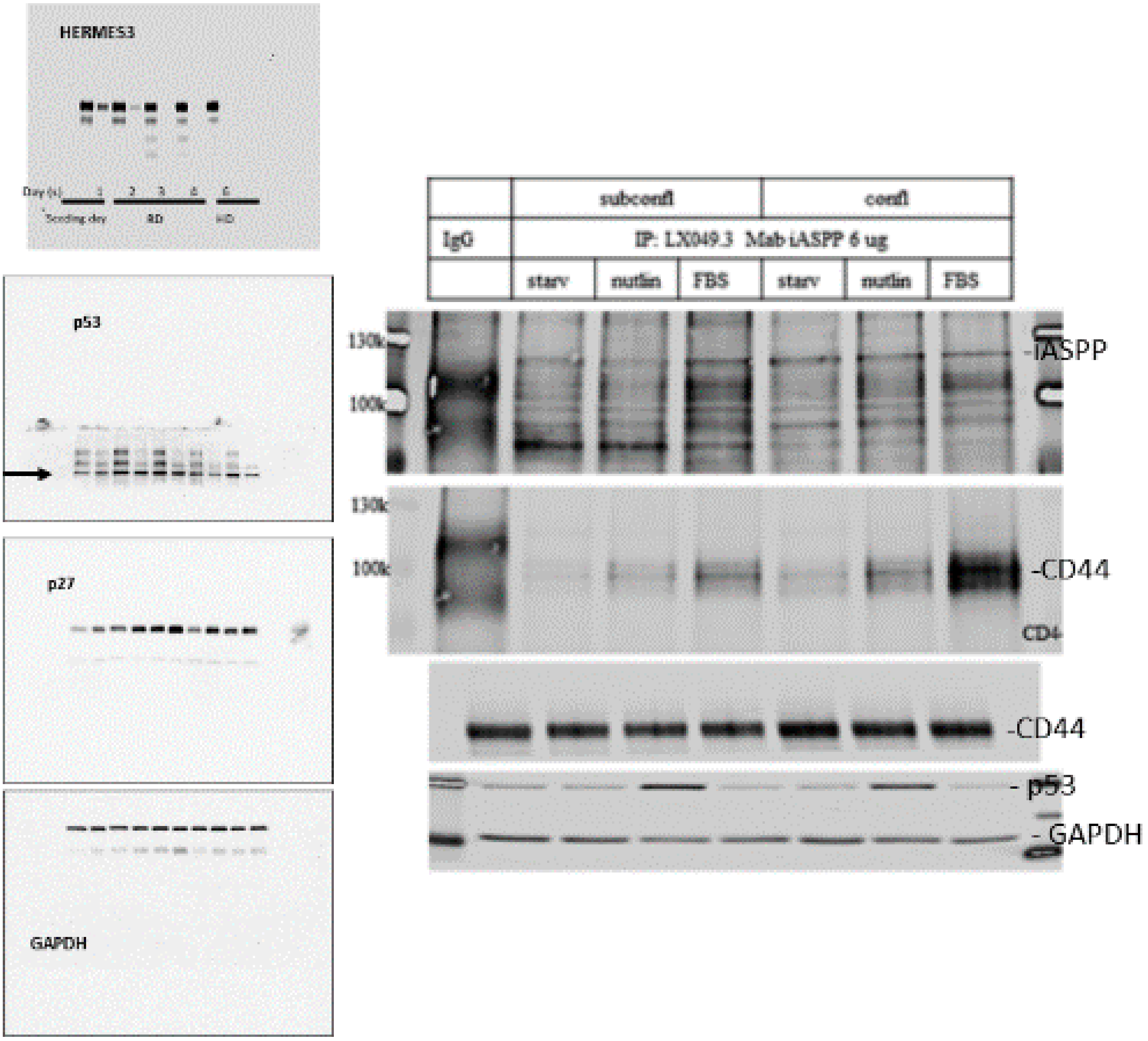

Uncropped blots corresponding to Figure 6C and 6E

Hyaluronan	–	–	+	+	–	–	–	–	+	+	–	–
PDGFBB	–	–	–	–	+	+	–	–	–	–	+	+
Hermes1	–	–	–	–	–	–	+	+	+	+	+	+
si iASPP	–	+	–	+	–	+	–	+	–	+	–	+

IB: iASPP

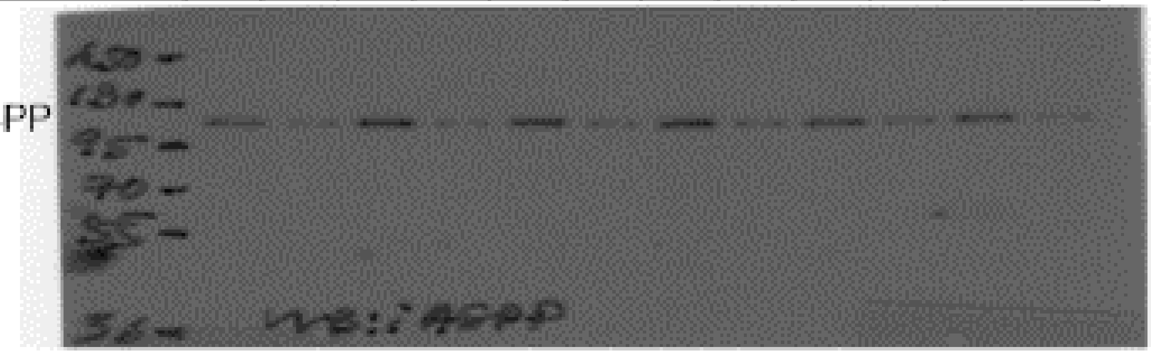

IB: GAPDH

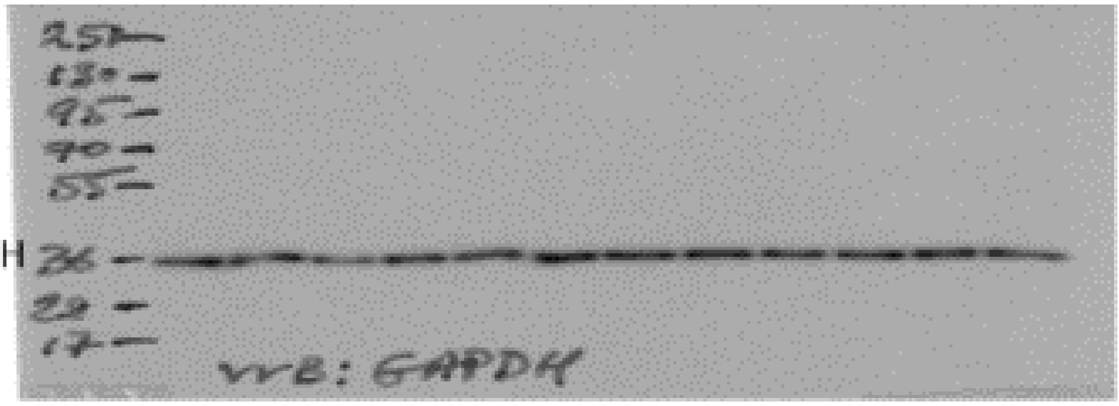

Uncropped blots corresponding to Figure 7

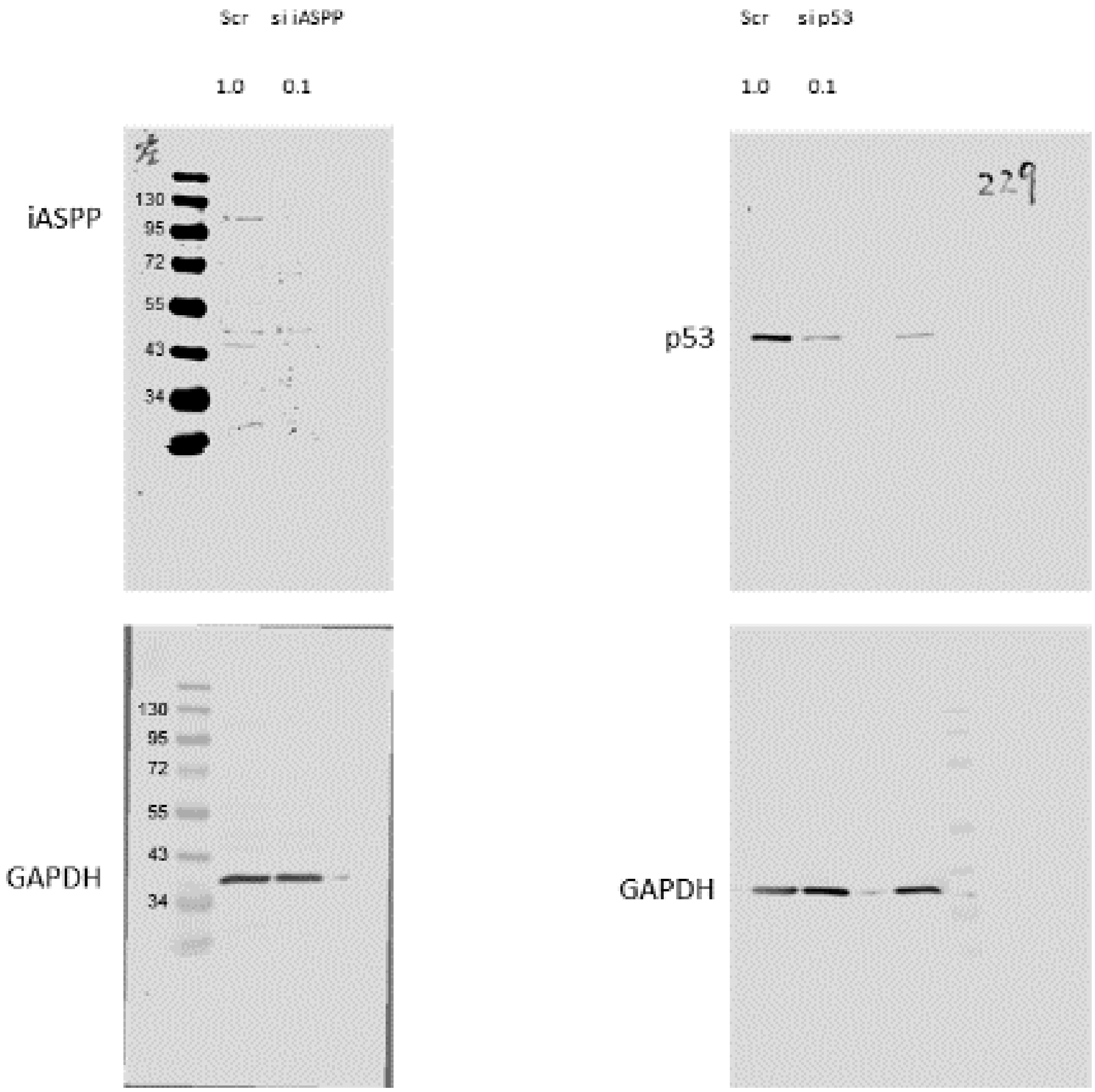

Uncropped blots corresponding to Figure 8
