# Supplementary material for: Hyaluronan-Induced CD44-iASPP Interaction Affects Fibroblast Migration and Survival
Source: Cancers (Basel). 2023 Feb 8;15(4):1082. doi: 10.3390/cancers15041082 (PMC9954134; doi:10.3390/cancers15041082)
Supplement: Supplementary file 1 [file cancers-15-01082-s001.zip › Supplementary Figures S1-S5.pdf]

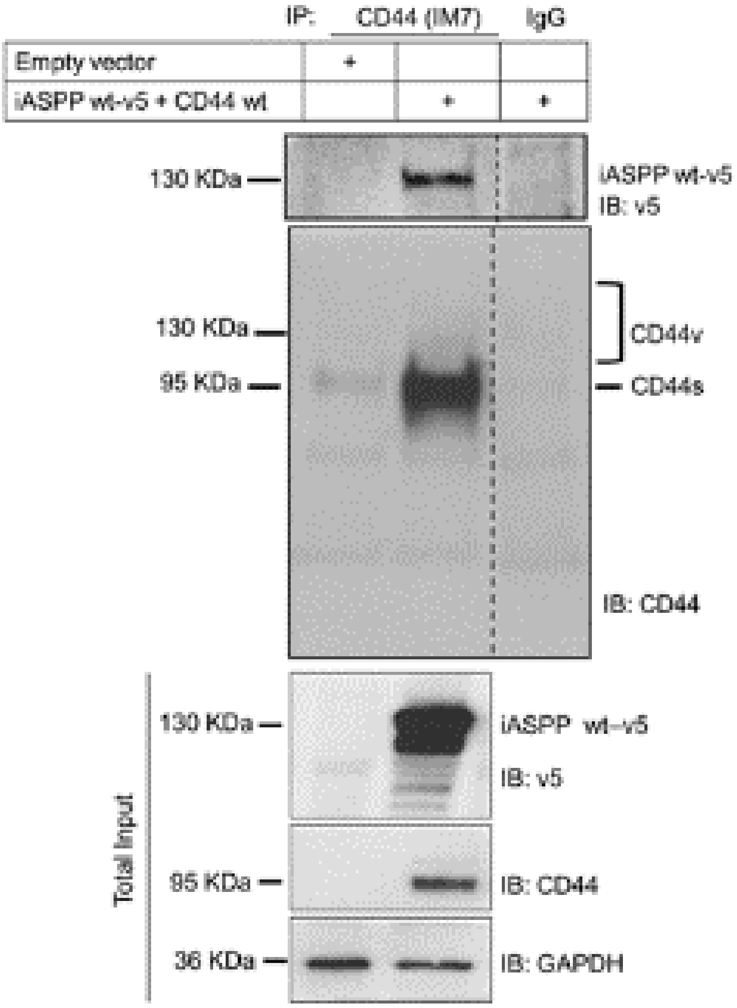

Figure S1: CD44 forms a physical complex with iASPP. v5-tagged wild type (iASPP wt-v5) and CD44 wt were ectopically co-transfected in HEK293 cells. Cell lysates were subjected to immunoprecipitation with an anti-CD44 antibody (IM7) and IgG isotype control, followed by immunoblotting with anti-CD44 (Hermes 3) and anti-V5 antibodies, confirming a complex between iASPP and CD44.

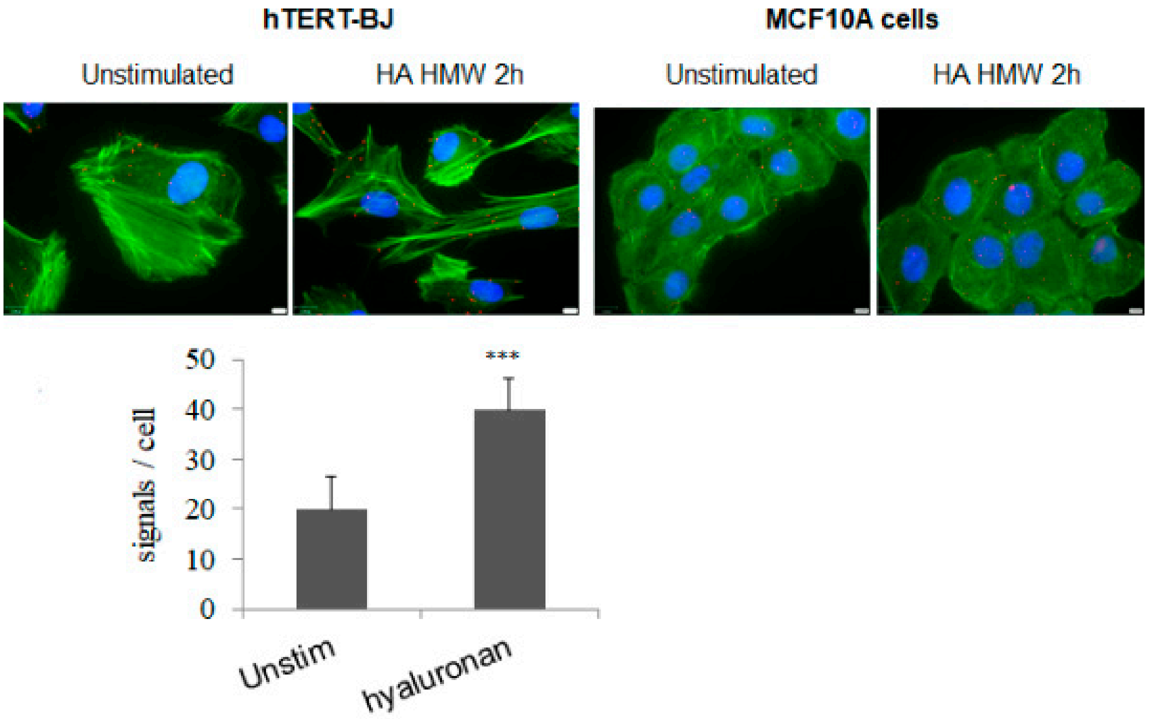

Figure S2: HMW HA stimulates CD44-iASPP complex formation *in situ*. Endogenous complexes between CD44 and iASPP in mesenchymal (hTERT-BJ and epithelial cells (MCF10) were confirmed by *in situ* proximity ligation assay (PLA). HMW HA (100 µg/ml) stimulation for two 2 h follows by PLA analysis increased the endogenous complexes between CD44 and iASPP in hTERT-BJ but not in MCF10A cells.

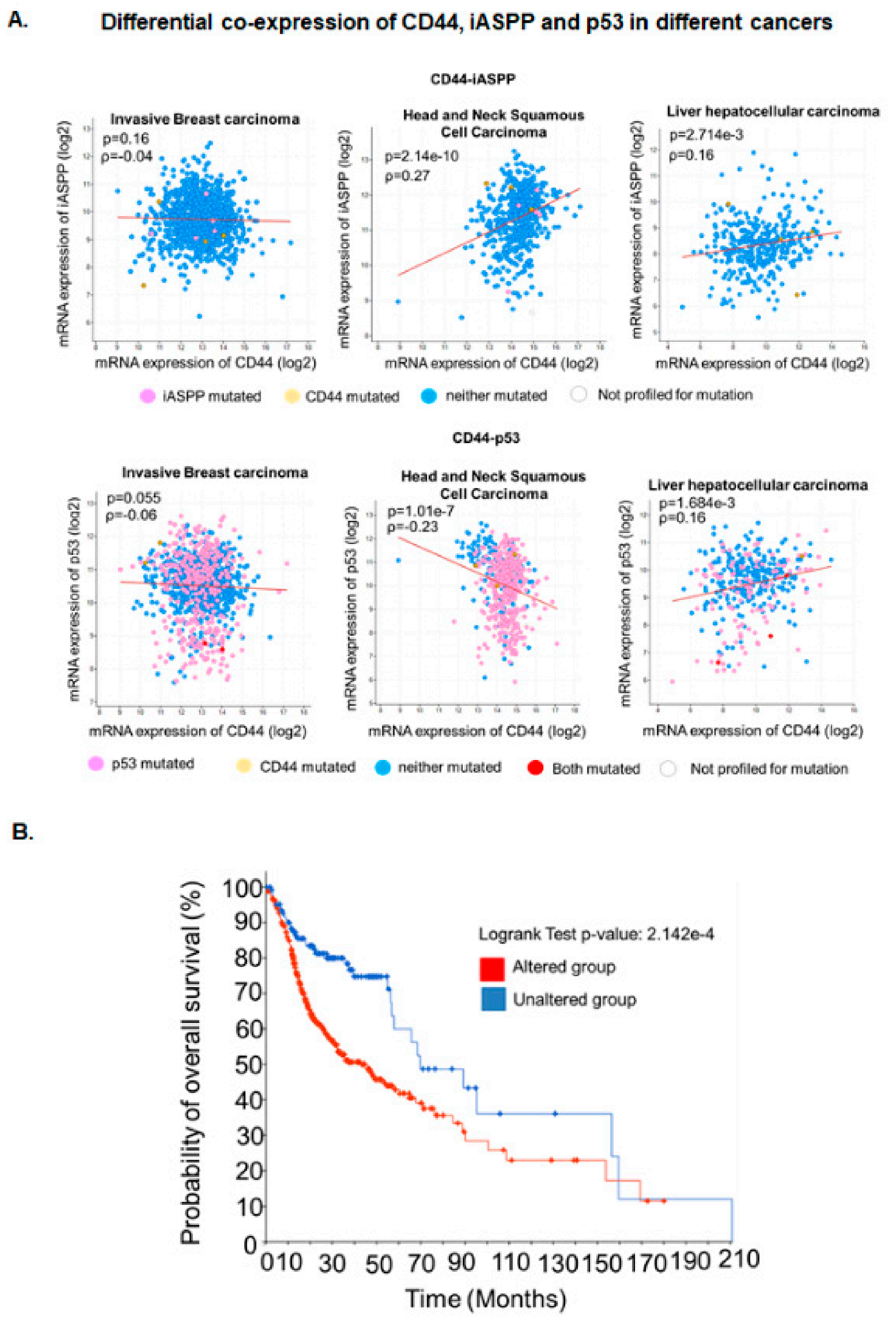

Figure S3: Correlation of *CD44*, *iASPP* and *p53* affect cancer survival: (A) In silico analysis of cancer patients' gene expression data from the TCGA database in cBioPortal shows differential co-expression of CD44-iASPP and CD44-p53 in three different types of cancer (B). Kaplan-Meier curves showing that unaltererd co-expression of *CD44*, *iASPP* and *p53* genes is correlated to increased cancer survival of head and neck squamous cell carcinoma patients compared to altered counterparts (i.e., upregulation or downregulation, with expression z-scores >2 or <-2).

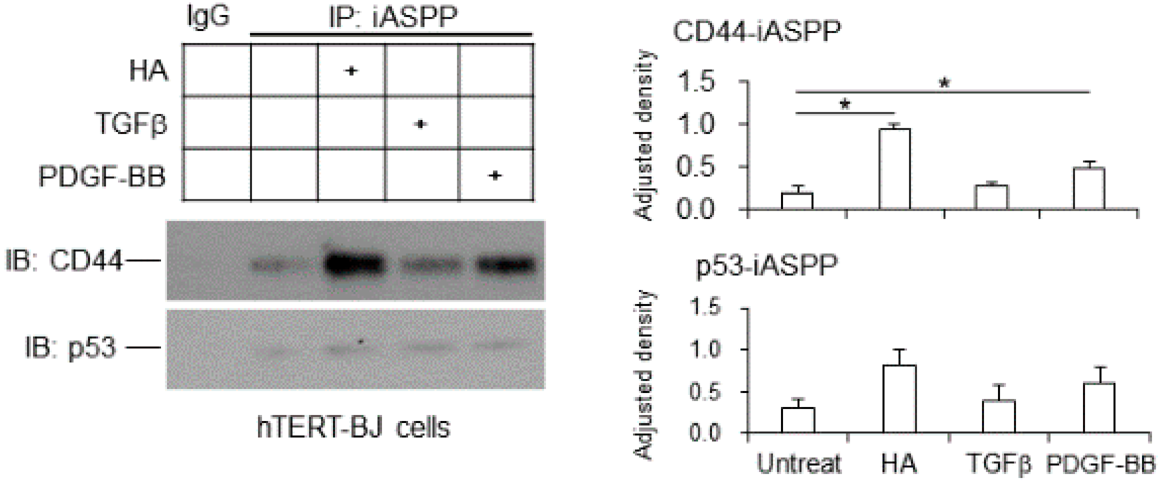

Figure S4: Effect of external stimuli on the formation of CD44-iASPP and p53-iASPP complexes. AG1523 cells were stimulated with, or not, hyaluronan (HA), TGFβ and PDGF-BB. Cell lysates were subjected to immunoprecipitation with an iASPP antibody (6 ug/ml), followed by immunoblotting with antibodies against CD44 and p53.

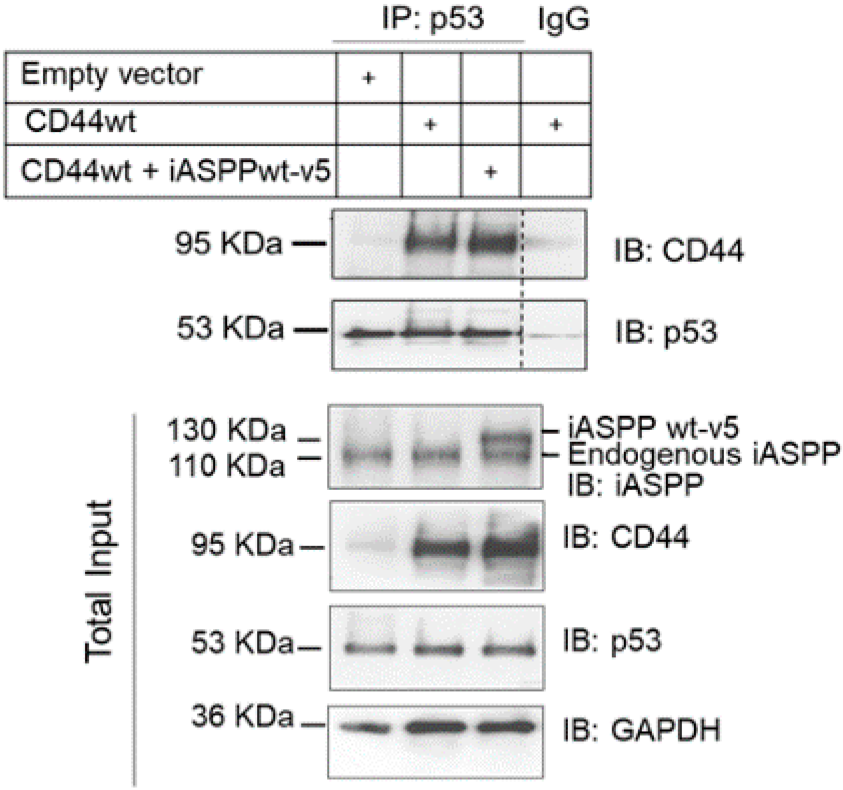

Figure S5: CD44 forms complex with p53: v5-tagged iASPP wt (iASPP wt-v5) and CD44 wt) were ectopically co-transfected in HEK293 cells and immunoprecipitated with an anti-p53 antibody and IgG isotype control, followed by immunoblotting with anti-CD44 (Hermes 3) and anti-p53 antibodies confirming a complex between CD44 and p53.
