# Supplementary material for: Hyaluronan-Induced CD44-iASPP Interaction Affects Fibroblast Migration and Survival
Source: Cancers (Basel). 2023 Feb 8;15(4):1082. doi: 10.3390/cancers15041082 (PMC9954134; doi:10.3390/cancers15041082)
Supplement: Supplementary file 1 [file cancers-15-01082-s001.zip › Table S1_Antibodies revised proofs.pdf]

Antibodies	Use	Supplier	Catalog Number	Dilution or Concentration
**Primary Antibodies**				
Anti-IgG mouse	IP	Sigma Aldrich	I5381<br>SLBQ7079V	6 µg
Anti- IgG Rabbit	IP	Sigma Aldrich	I5006	3 µg
Anti-mouse iASPP, LX49.3	IP / IB	Sigma Aldrich	A4605<br>035M4783V	6 µg<br>1:1000
Anti-rabbit iASPP	IP	Sigma Aldrich	A4605<br>035M4783V	6 µg
Anti-iASPP	IP / IB	Abcam	ab34898	3 µg
Anti-CD44, Hermes3	IB	Professor Sirpa Jalkanen	---	1:10 000
Anti-CD44, Hermes1	blocking	Professor Sirpa Jalkanen	---	20 µg
Anti-CD44, IM7	IP / IB	Santa cruz	SC-18849	1:200
Anti-p53 DO1	IB	Santa cruz	Sc-126	1:1000
Anti-p-ERK	IB	Cell Signaling Technology	#4370	1:1000
Anti-ERK	IB	Cell Signaling Technology	#4695	1:1000
Anti-p-AKT	IB	Cell Signaling Technology	#4060	1:1000
Anti-AKT	IB	Cell Signaling Technology	#2920	1:1000
Anti-p-p38	IB	Cell Signaling Technology	#9211	1:1000
Anti-GAPDH	IB	Cell Signaling Technology	#5174	1:20000
Anti-p-Merlin	IB	Cell Signaling Technology	#13281	1:1000
Anti-Merlin	IB	Cell Signaling Technology	#6995	1:1000
Anti-V5	IB	BioSite	MCA1360A	1:1000
Anti-Laminin	IB	Cell Signaling Technology	#2032	1:1000
Anti-p-S6	IB	Cell Signaling Technology	#5364	1:1000
Anti-Total S6	IB	Cell Signaling Technology	#2317	1:1000

Anti-p27	IB	Cell Signaling Technology	#2552	1:1000
**Secondary Antibodies**				
HRP anti mouse; Light chain specific	IB	Jackson Immuno-research	115-035-174	1:10000
HRP goat anti-rabbit IgG	IB	Thermo Fisher Scientific	# 65-6120	1:10000
HRP goat anti-mouse IgG	IB	Thermo Fisher Scientific	# 65-6120	1:10000
