# Supplementary material for: Hyaluronan-Induced CD44-iASPP Interaction Affects Fibroblast Migration and Survival
Source: Cancers (Basel). 2023 Feb 8;15(4):1082. doi: 10.3390/cancers15041082 (PMC9954134; doi:10.3390/cancers15041082)
Supplement: Supplementary file 1 [file cancers-15-01082-s001.zip › Table S2_In_Silico.pdf]

**Table S2.** ***In silico*** **prediction of possible interaction of CD44 with iASPP C-terminal domain**

| **CD44 -iASPP interaction** | **Predicted score** | | | **Combined Score** | **Comment** |
|---|---|---|---|---|---|
| | **Based on sequence similarities to known interacting protein pair** | **Based on statistical propensities of domain-domain interactions** | **Based on sum of edge weights along the shortest path between homologous proteins in a protein-protein interaction network** | | |
| CD44 FL vs. iASPP FL (1-828 aa) | 0.8130 | 0.3897 | 0.8351 | **0.7394** | Possible strong interaction |
| CD44 FL vs. iASPP n-terminus (1-478 aa) | 0.3537 | 0.0000 | 0.0000 | 0.3537 | Possible weak interaction |
| CD44 FL vs. iASPP c-terminus (479-828 aa) | 0.8130 | 0.3897 | 0.8351 | **0.7394** | Possible strong interaction |

FL: Full length; aa: amino acid
