# Supplementary material for: Hyaluronan-Induced CD44-iASPP Interaction Affects Fibroblast Migration and Survival
Source: Cancers (Basel). 2023 Feb 8;15(4):1082. doi: 10.3390/cancers15041082 (PMC9954134; doi:10.3390/cancers15041082)
Supplement: Supplementary file 1 [file cancers-15-01082-s001.zip › Table S3.pdf]

Table S3: Correlation and co-expression of CD44, iASPP and p53 in cancer patients

Cancer Type	Head and Neck Squamous Cell Carcinoma	Adult soft tissue sarcoma	Invasive Breast carcinoma	Lung squamous cell carcinoma	Liver hepatocellular carcinoma	Glioblastoma multiforme	Kidney Renal Papillary Cell Carcinoma
Sample size*	528	206	1084	487	372	585	283
**Survival analysis involving mRNA expression of CD44, iASPP & p53**							
Difference between survival curve with altered mRNA expression vs. unaltered mRNA expression (Log rank Test p-value)	**2.142e-4**	0.89	**0.04**	0.23	**0.01**	**7.806e-3**	**0.03**
Survival rate high for mRNA expression level	Unaltered	No significant difference	Unaltered	No significant difference	Unaltered	Altered	Unaltered
**CD44-iASPP**							
Epistatic relationship	Mutual exclusivity	Co-occurrence	**Mutual exclusivity**	Co-occurrence	**Co-occurrence**	Mutual exclusivity	Co-occurrence
	NS (p=0.48)	NS (p=0.63)	**S (p=0.02)**	NS (p=0.28)	**S (p=0.02)**	NS (p=0.82)	NS (p=0.38)
Co-expression	**PC; ρ=0.27**	NC; ρ=-0.02	NC; ρ=-0.04	PC; ρ=0.19	PC; ρ=0.16	PC; ρ=0.44	PC; ρ=0.11
	**p=2.14e-10**	p=0.79	p=0.16	**p=3.015e-5**	**p=2.714e-3**	**p=2.14e-10**	p=0.06
**CD44-p53**							
Epistatic relationship	Mutual exclusivity	Co-occurrence	Mutual exclusivity	Mutual exclusivity	**Co-occurrence**	Co-occurrence	Co-occurrence
	NS (p=0.31)	NS (p=0.50)	NS (p=0.34)	NS (p=0.41)	**S (p=0.01)**	NS (p=0.21)	NS (p=0.50)
Co-expression	**NC; ρ=-0.23**	NC; ρ=-0.03	NC; ρ=-0.06	PC; ρ=-0.03	PC; ρ=0.16	PC; ρ=0.08	PC; ρ=0.02
	**p=1.01e-7**	p=0.0718	p=0.055	p=0.45	**p=1.684e-3**	p=0.29	p=0.74

[Note]: S = significant; NS = not significant; *source: TCGA database; PC = positive correlation; NC = negative correlation; ρ= Spearman's rank correlation co-efficient; p = p-value
